# Supplementary material for: Integrated Microfluidic Platform for High‐Throughput Generation of Intestinal Organoids in Hydrogel Droplets
Source: Adv Sci (Weinh). 2026 Jan 5;13(13):e16507. doi: 10.1002/advs.202516507 (PMC12955914; doi:10.1002/advs.202516507)
Supplement: Supplementary file 1 — Supporting File 1: advs73411‐sup‐0001‐SuppMat.docx. [file ADVS-13-e16507-s003.pdf]

Supplementary Information:

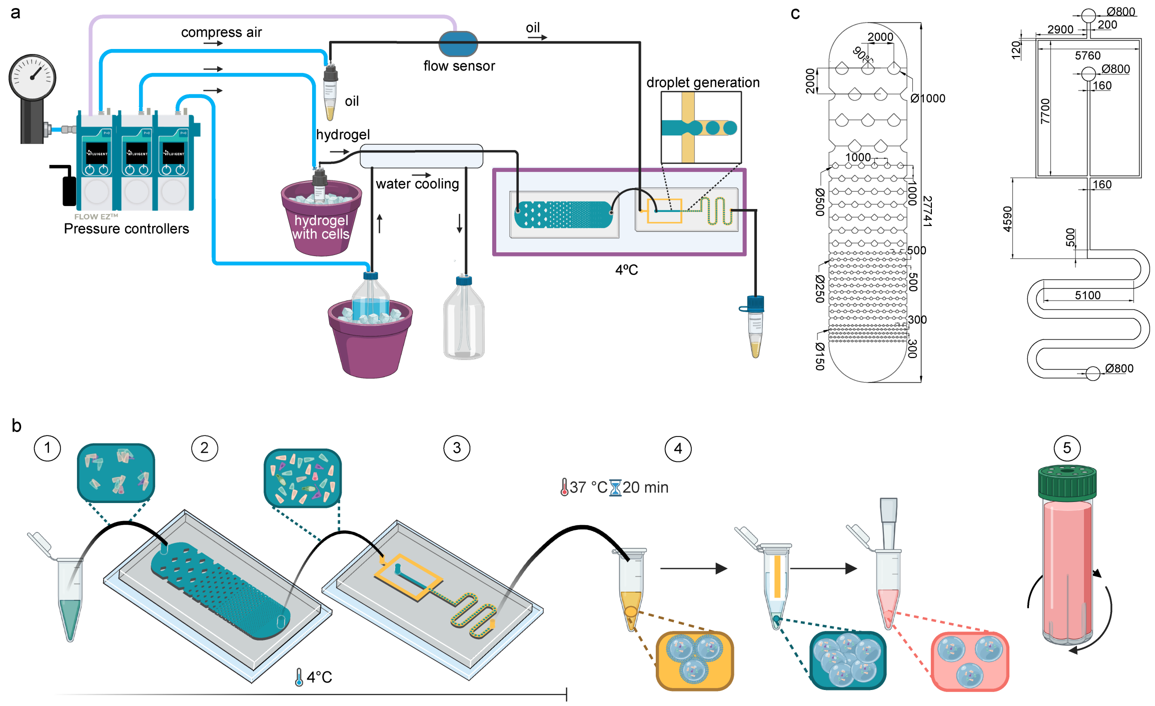

Supplementary Figure 1: a) Schematic overview of microfluidic set up for droplet generation. b) A schematic of the system workflow. Clumping cell hydrogel suspension (1) is homogenized through the cell distribution device (2) and connected to the flow-focusing encapsulation device (3). Formed hydrogel droplets undergo polymerization, oil extraction and resuspension into media (4). Cell-hydrogel particles are cultured in a bioreactor (5). c) Technical drawings of dissociation chip and flow-focusing device. Schematics were partially created with BioRender.com

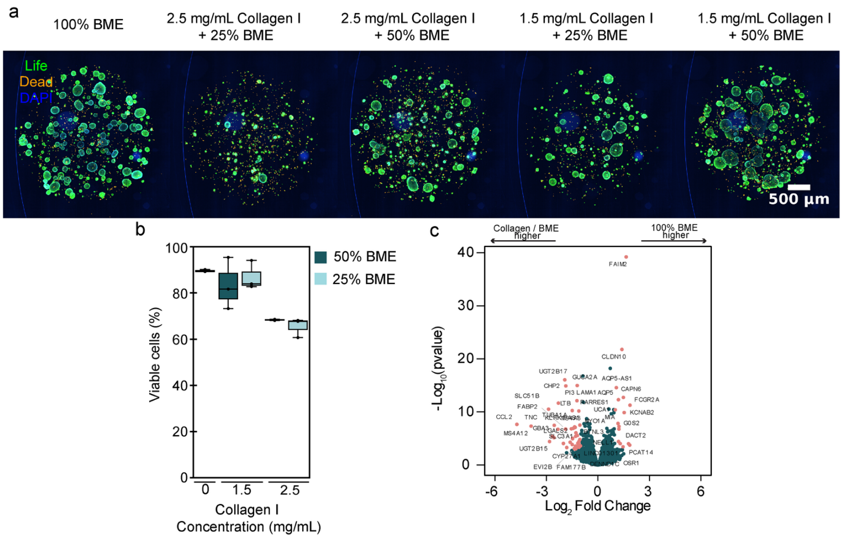

Supplementary Figure 2: a) Representative fluorescence images of live/dead staining of organoids growth at different Collagen I / BME (Cultrex) ECM at day 7 (scale bar 500 µm). b) Quantitative fluorescence analysis of cell viability in intestinal organoids at days 7 of culture in domes composed of different ECM by live/dead staining ($n_{samples} = 3$). c) Volcano plot of differentially expressed genes between 1.5 mg/mL Collagen–25% BME (Matrigel) ECM mix (left) and BME (Matrigel) (right) subsets ($p < 0.05$, log2 Fold Change > 1).

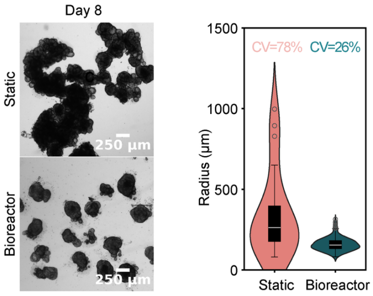

Supplementary Figure 3: Representative brightfield images of droplets cultured in 6 well plate or bioreactor (scale bar 250 µm). Size analysis of organoids grown in droplets in static condition or in bioreactor on day 8 with coefficient of variation of organoid size highlighted above (n_{static} = 22, $n_{bioreactor}$= 237).

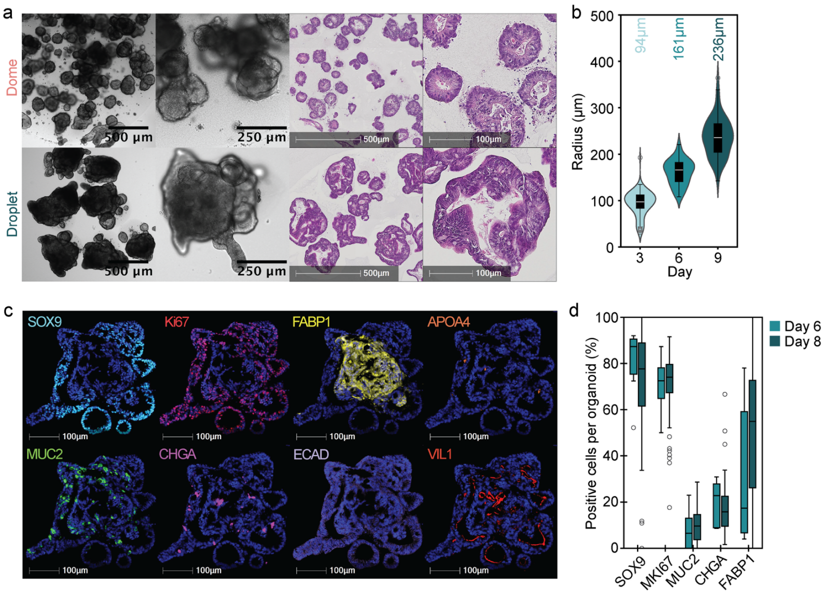

Supplementary Figure 4: a) Brightfield images and H&E staining comparison of duodenum organoids in a bioreactor on day 8 grown in domes or hydrogel droplets. b) Size development analysis of organoids in droplets over several days, with average size highlighted above ($n_{organoids}$ ≥ 55). c) mIF staining of organoids in hydrogel droplets in a bioreactor on day 8. d) Quantification of mIF staining of organoids in droplets on day 6 and day 8 ($n_{organoids}$ ≥ 17).

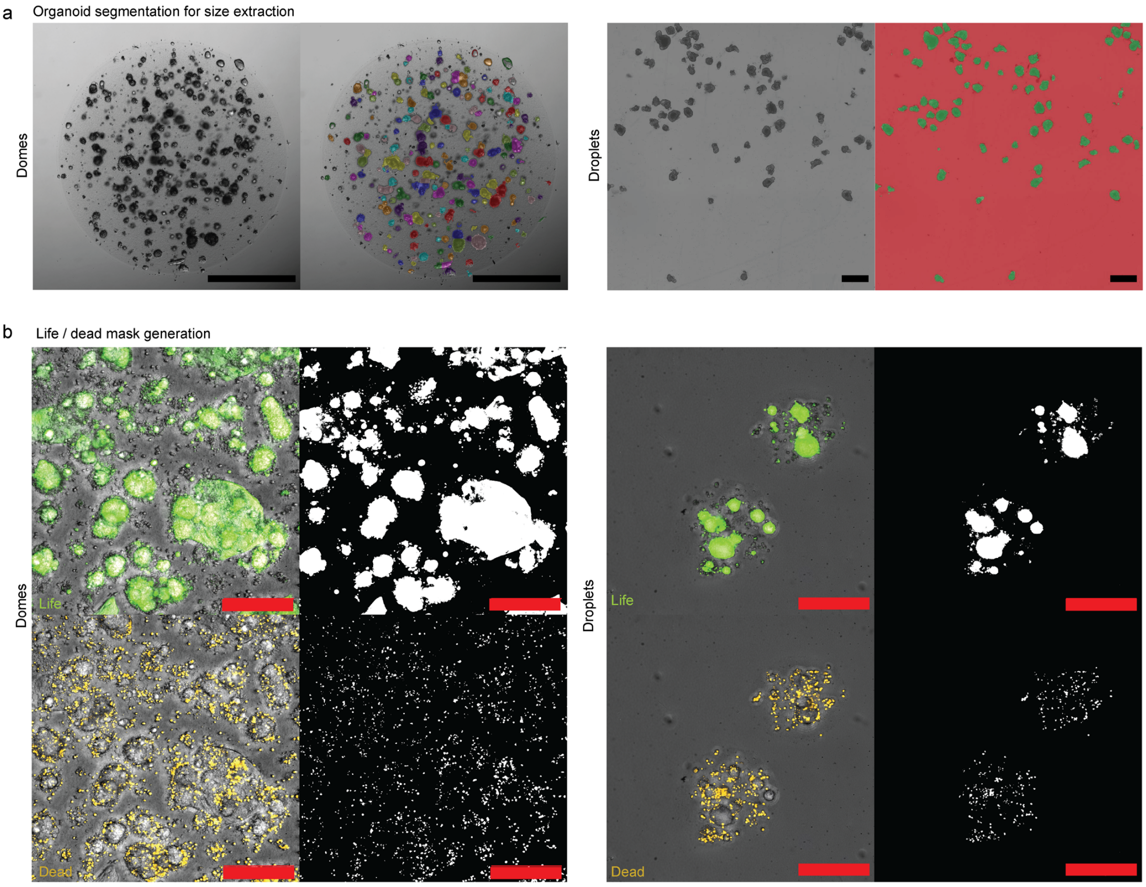

Supplementary Figure 5: a) Representative images of organoid size analysis. Brightfield image of dome with corresponding color-coded image segmentation masks for individual organoids on left, and brightfield image of organoids within droplets with corresponding green image segmentation masks for individual organoids on right (scale bars 1 mm). b) Representative images of cell viability analysis, overlay brightfield and fluorescence image with the corresponding segmentation mask. Life cells stained by Calcein AM are shown in green and dead cells stained with ethidium homodimer-1 are shown in orange (scale bars 200 µm).

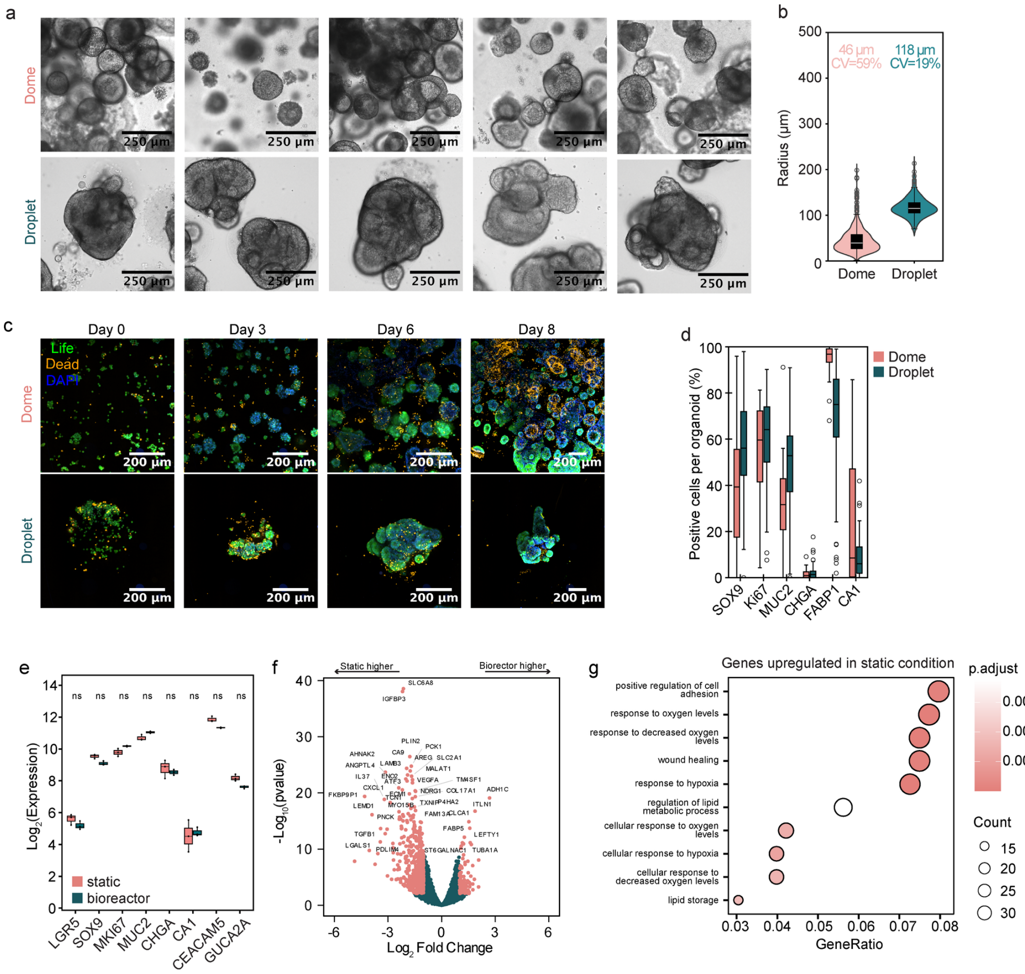

Supplementary Figure 6: a) Brightfield images of biological replicates of colon organoids in a bioreactor on day 8 grown in domes or hydrogel droplets. b) Size analysis of organoids grown in domes or droplets on day 6 with coefficient of variation of organoid size highlighted above ($n_{\text{organoids}} \geq 90$) c) Representative fluorescence images of live/dead staining of organoids growth in domes or droplets on days 0, 3, 6, 8 (scale bar 200 µm). d) Quantification of mIF staining of organoids in domes or droplets in a bioreactor on day 8 ($n_{\text{organoids}} \geq 67$). e-g) Bulk RNA sequencing of organoids grown in droplets in static condition or in bioreactor on day 8 (3 technical replicates). e) Quantification of log2 transformed expression levels. Statistical significance between conditions was assessed using the Wilcoxon test, with *p*-values adjusted for multiple testing using the Benjamini-Hochberg method. f) Volcano plot of differentially expressed (DE) genes between static (left) and bioreactor (right) condition ($p < 0.05$, log2 Fold Change > 1). g) Plot showing significantly enriched Gene Ontology biological processes of genes in colon organoids grown in static condition (*p*-value < 0.05, *q*-value < 0.2).

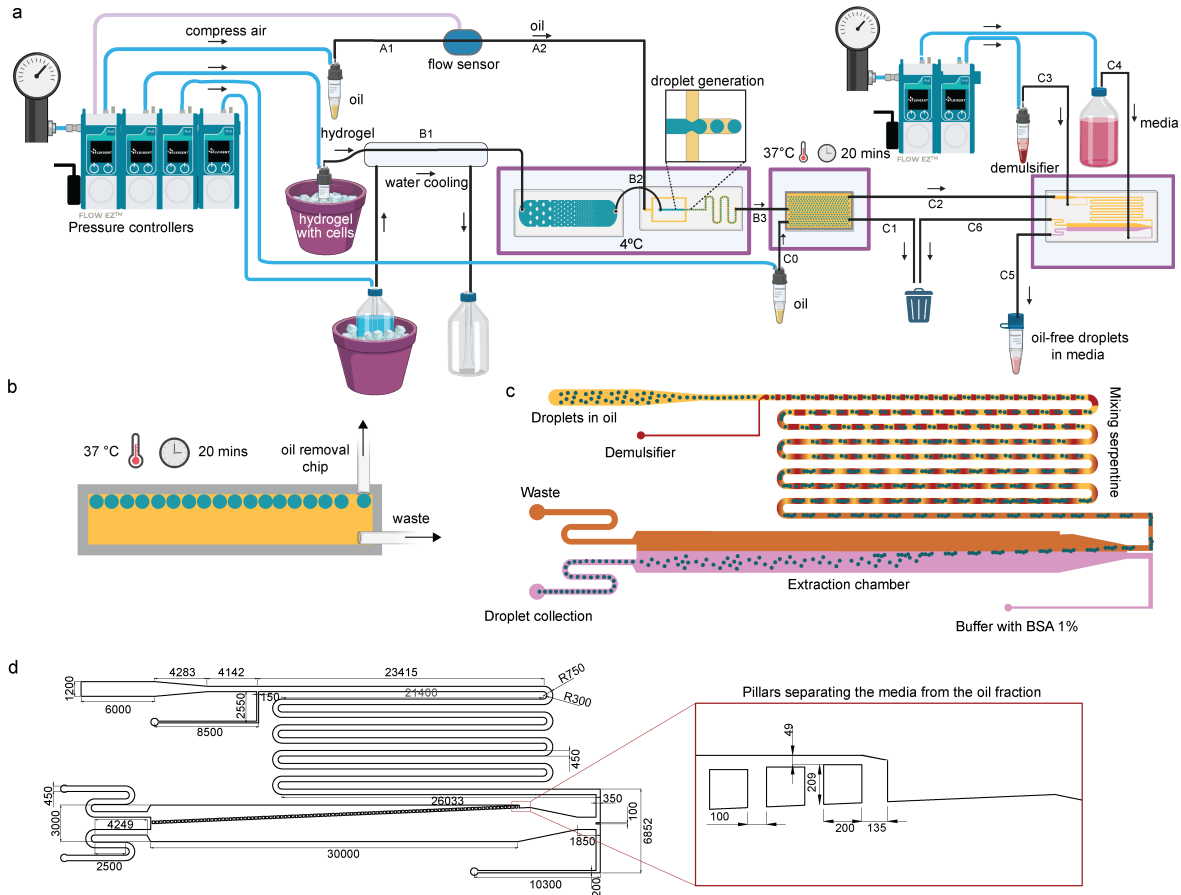

Supplementary Figure 7: Oil removal microfluidic device: a) Schematic overview of microfluidic set up for droplet generation integrated with polymerization and oil removal module. Schematics were partially created with BioRender.com b) A detailed schematic of the polymerization chamber. c) A detailed schematic of the oil removal chip with workflow description. Schematics were partially created with BioRender.com d) Technical drawings of the flow focusing device.

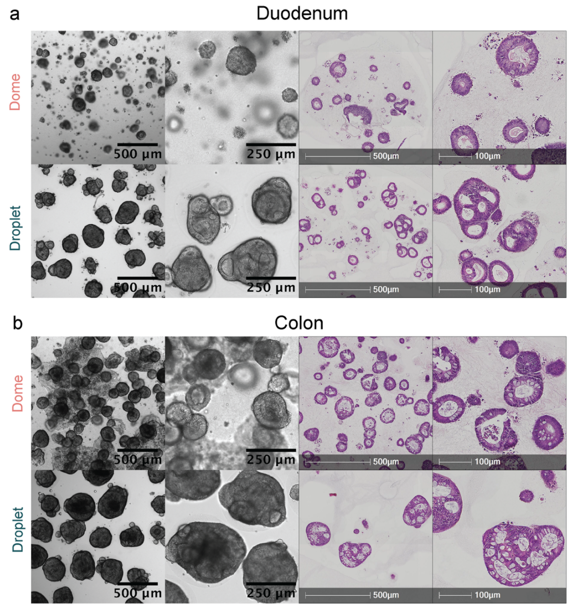

Supplementary Figure 8: Brightfield images and H&E staining comparison of organoids in a bioreactor on day 8 grown in domes or hydrogel droplets generated with the oil removal device: a) organoids derived from duodenum, b) organoids derived from colon (scale bars as indicated 500 µm, 250 µm, 100 µm).

Supplementary table 1: Key resource table.
